# Stretchable, Multi-Layered Stack Antenna for Smart/Wearable Electronic Applications

**DOI:** 10.3390/ma15093275

**Published:** 2022-05-03

**Authors:** Kiwoong Hong, Jonam Cho, Gunchul Shin

**Affiliations:** School of Materials Science and Engineering, University of Ulsan, 12 Technosaneop-ro 55 beon-gil, Nam-gu, Ulsan 44776, Korea; kiwoonghong@naver.com (K.H.); dyfska@naver.com (J.C.)

**Keywords:** wireless, NFC, PDMS, antenna, wearable, stretchable

## Abstract

The development of microelectronics has been achieved by improving its performance through miniaturization. This was possible through the development of silicon-based semiconductor process technology, but recently, the demand for wearable or flexible devices has increased. These devices are made using various functional elements based on materials that are difficult to utilize with semiconductor devices that contain existing hard silicon-based materials and are bent or flexibly stretched. In this study, wireless antennas suitable for wearable devices were implemented in a stretchable form. It was possible to stably receive a wireless signal, even with a strain of 20% or more, and power light-emitting diodes (LEDs), microheaters, etc. By devising a multi-layered stack antenna without the existing semiconductor process, it was possible to improve the antenna’s reception performance. It is expected that this can be applied in various ways to smart wireless sensors and wearable biomedical devices using the near-field communication (NFC) of smartphones.

## 1. Introduction

The development of semiconductor devices that are based on silicon materials has continued for decades. Silicon is a readily available material that can be implemented in the form of a single crystal wafer more easily than other materials. Moreover, the desired characteristics of a semiconductor material can be realized through doping. However, silicon with good crystallinity is limited in that it is difficult to use in bendable devices or wearable electronics because it is hard and easily broken owing to the characteristics of the material. To solve this problem, silicon was processed into a ribbon with a nanometer-scale thickness to form a flexible or stretchable device; however, there were restrictions on the production of a nanoribbon from a special wafer such as a silicon-on-insulator (SOI) wafer or using an anisotropic etching, etc. [1,2,3,4,5]. Recently, devices that can be stretched while using existing commercial rigid chips have been developed, and batteries or wireless antennas have been used in wearable applications [6,7,8,9,10]. These devices have sufficiently ensured various functions and stretching characteristics, but the existing silicon semiconductor process technology proved to be difficult to use, so these applications were mainly composed of single-layer antennas and circuits. Accordingly, their wireless receiving performance was limited. The applications varied depending on the frequency of their use as well as the size of the receiving antenna, and an ultra-high frequency (UHF) above GHz was transmitted far away but it was also heavily influenced by directionality and its surrounding conducting materials. NFC-based wireless antennas were relatively less affected by their surroundings but had the disadvantage of a few centimeters [11,12,13,14,15,16]. This research was able to power light-emitting diodes (LEDs) and a micro-heater by receiving wireless signals at distances of several tens of centimeters, which was sufficient for various applications, even at the small size of 1.6 cm × 1.6 cm, through multilayered stack antennas. In addition, it was possible to exclude the existing clean room-based semiconductor process, to manufacture a device with a multi-layered wireless antenna capable of stretching through a simple patterning process, and to confirm the improvement of the antenna performance. This technology can be applied to flexible and stretchable devices that are difficult to produce with existing silicon-based parts and that can be used to produce smart and wearable devices.

## 2. Materials and Methods

**Figure 1:**
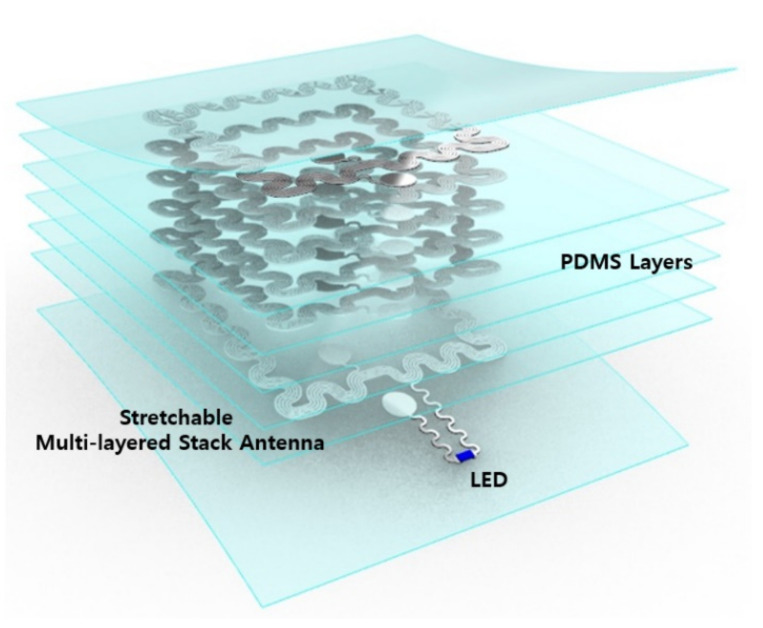
Schematic illustration of the stretchable, multi-layered stack antenna device.

**Figure A1:**
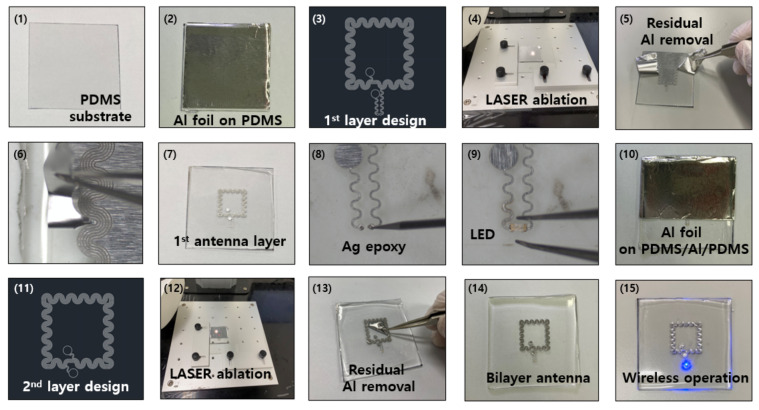
Photographs of detailed fabrication process for stretchable, multi-layered antenna devices.

Stretchable multi-layered stack-antenna elements were created with polydimethylsiloxane (PDMS), one of the most widely used flexible materials [6,7,17,18,19]. PDMS (Sylgard 184, Dow Corning, Midland, MI, USA) was manufactured in the form of a thin film through spin-coating on a PET-based polymer film that was easy to detach afterwards. Aluminum foil (thickness: 15 μm) was attached to it, and the antenna layout of the first layer was patterned with LASER marker equipment (Hyosung Laser, Bucheon, Korea) [17,18,19]. The unnecessary aluminum was removed, and the PDMS was again spin-coated after soldering the LED. Using the same method, the antenna on the second layer was also made of aluminum by patterning with LASER equipment, and up to six layers were manufactured in the same manner. The antenna between the layers was finished using a micro-punching tool or by removing the PDMS part on the connection pad and attaching it using Ag-conductive epoxy (Silver conductive epoxy 8330, MG chemicals, Ontario, Canada). The shape of the device is shown in Figure 1, and a device consisting of an antenna of up to six layers was manufactured with a maximum thickness of 300 μm or less; the detailed fabrication process can be seen in Figure A1.

## 3. Results and Discussions

### 3.1. Stretchable, Multi-Layered Stack Antenna

**Figure 2:**
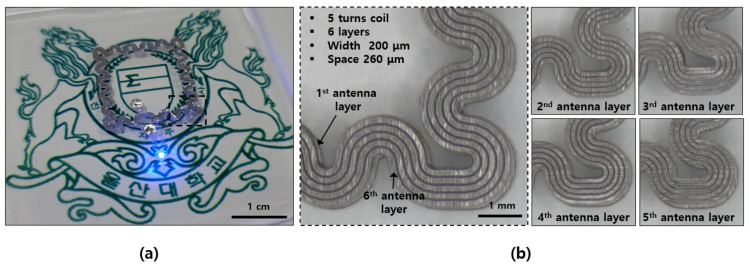
Photographs of (**a**) wireless operation of optoelectronic device with multi-layer antenna and (**b**) stretchable antenna layers.

The completed device is shown in Figure 2a, and it is evident that the LED was driven wirelessly using the power received from the multi-layered wireless antenna. A wireless antenna with an outer diameter of 1.6 cm × 1.6 cm was implemented in a serpentine form to allow stretching, and five turns of coil per layer were implemented with a width of 200 μm and at an interval of 260 μm. While raising the multi-layered antenna from the first to the sixth layer, the serpentine antenna was patterned by aligning it with the lower layer as much as possible. The antenna pattern for each layer is shown in Figure 2b. To maximize the performance of the receiving antenna, the outer diameter of the antenna can be increased, which increases the size of the entire device while possibly limiting its wearability. To improve the performance of the receiving antenna without increasing its size and reducing its inner diameter, a multilayer antenna was applied in this study. Using the existing semiconductor process, equipment such as an evaporator or sputter equipment for thin metal film deposition, plasma-enhanced chemical vapor deposition (PECVD) equipment for insulating film deposition, and a mask aligner for patterning were required. A device that can be manufactured in a non-cleanroom circumstance was developed using only simple equipment such as a LASER marker for patterning and Ag paste for interlayer connection. Unlike cleanroom-based semiconductor processes, such as high temperature and high vacuum processes that usually take several days or more, the process of manufacturing a multilayer device in the atmosphere may be completed within a few hours, thereby having an advantage in terms of time and convenience [20].

### 3.2. Fabrication and Connection of via

**Figure 3:**
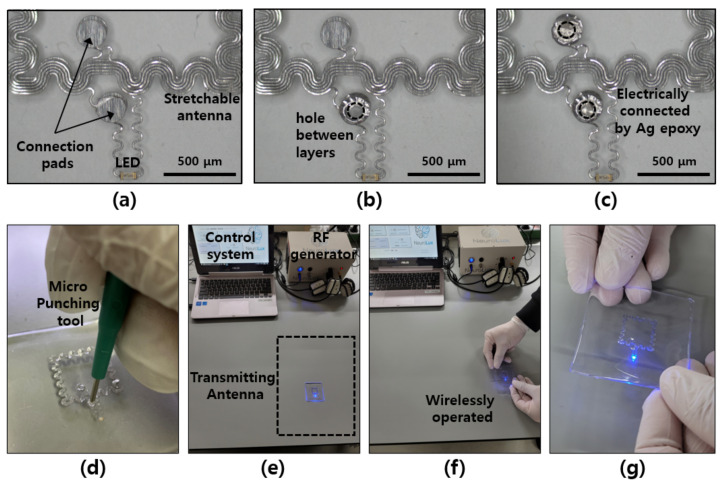
Images of optoelectronic device with multi-layered antenna. (**a**–**c**) microscope images of fabrication and connection process for via; (**d**) micro-punching tool; (**e**) wireless power transfer system; (**f**,**g**) wireless operation of fabricated optoelectronic device.

In order to construct a multilayered antenna, a new process for inter-layer connection was developed. Building a semiconductor via the traditional manufacturing process proceeds through thin film deposition, patterning using lithography, and etching, but here, the interlayer connection was easily made using a micro-punching tool and conductive epoxy. Figure 3a–c show the connection process of the double-layer antenna in sequence. The antenna pattern and connection pad stacked in two layers were completed using the micro-punching tool to make a hole, as shown in Figure 3d, filling the hole with Ag epoxy paste, and then heat-treating it at 100 °C. After PDMS spin coating, heat treatment was performed at 100 °C for 10 min on each layer for curing, which is the same as the Ag epoxy curing conditions, so no additional thermal damage or hardening occurred on either the PDMS or epoxy parts. The finished device was driven wirelessly through an NFC-based wireless power transmitting system (Neurolux, Chicago, IL, USA) [17,18,19]. After the 13.56 MHz radio frequency signal was generated by the rf signal generator, the signal was provided from the transmitting antenna installed under the desk through the control system (Figure 3e–g). Using a 30 cm × 40 cm transmitting antenna installed 3 cm below the desk surface, it was confirmed that the completed stretchable multilayer antenna and the optoelectronic device connected to it were operating.

### 3.3. Performances of Antenna as a Function of Number of Layers

**Figure 4:**
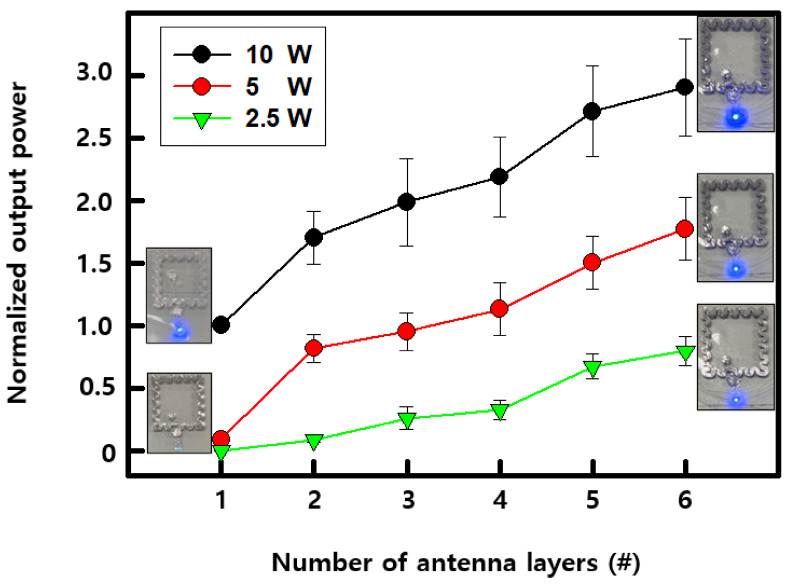
A graph of normalized output power of optoelectronic devices as a function of number of antenna layers with different transmitting powers.

**Figure A2:**
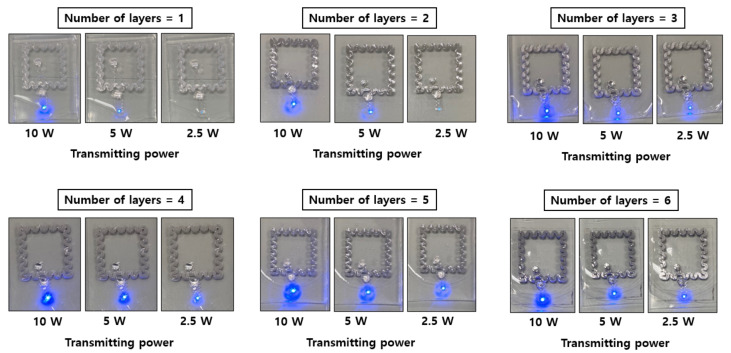
Photographs of wireless operated optoelectronic devices as a function of number of layers with different transmitting powers.

We confirmed that the receiving performance of the wireless antenna changed according to the increase in the antenna layers, based on the brightness of the LED driven by the receiving power. The output power of the LED was compared by manufacturing an antenna stacked from a single layer to up to six layers after increasing the power of the transmitting antenna twice, from 2.5 W to 5 W and then to 10 W. A serpentine antenna of 1.6 cm × 1.6 cm dimensions was used as the basic structure, and in the case of the single-layer antenna, the LED light was hardly visible at 2.5 and 5 W. The data were graphed in Figure 4 after normalizing the brightness at 10 W of a single-layer antenna to 1. As the number of layers increased, the brightness of the LED increased, and the brightness of the LED in the six-layer device was about three times (output power: ~28 mW) higher than that of the one-layer device with the same transmitting power. In the case of low power, the improvement was greater, with a brightness improvement of about 12 times based on 5 W and about 30 times based on 2.5 W. This was because the number of turns of the coil was doubled owing to the multilayer antenna; the cross-sectional part A of the coil increased up to three times, the resistance of the coil was reduced (R = ρL/A) because there was no change in the internal diameter, and the received voltage was amplified. Finally, the LED was driven sufficiently even at low transmitting power. According to the previously reported results, it is most effective to increase the size of the outer diameter of the antenna to maximize the wireless receiving performance [17,19,21]. This is achieved by increasing the number of turns of the coil or by lowering the resistance of the coil as long as it does not reduce the number of turns. Additionally, if a fine pattern is produced through a commercial semiconductor process, the performance of the antenna can be improved with narrower coil spacing. In summary, in order to improve the performance of the wireless receiving antenna, it is recommended to increase the outer and inner diameters of the antenna, to increase the number of turns or to reduce the resistance of the coil, and to minimize the spacing between the coils. In this work, it was possible to improve the receiving performance by configuring the multilayer antenna to double the number of turns and reduce the resistance by one-third without reducing the inner diameter. The image brightness of the LED driven by the receiving antenna was affected by changing the number of antenna layers, and the transmitting power can be further confirmed in Figure A2.

### 3.4. Stretchable and Bendable Performances

**Figure 5:**
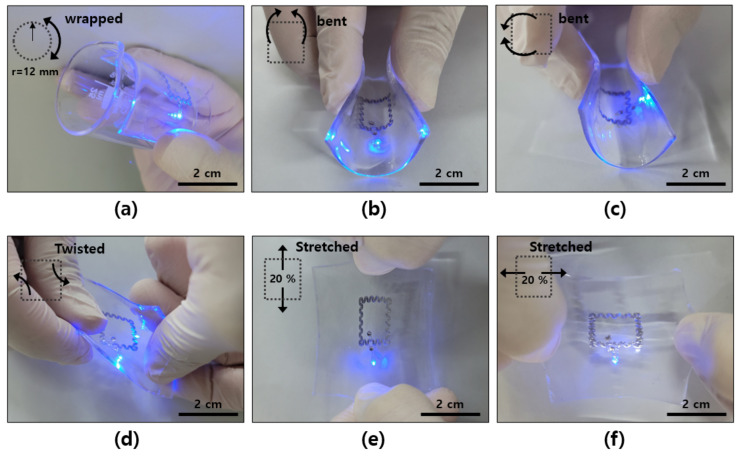
Stretchable and bendable performances of optoelectronic devices with a multi-layered antenna: (**a**) wrapping on a beaker with a radius of 12 mm; (**b**) bending in a lateral direction; (**c**) bending in a horizontal direction; (**d**) twisting in a diagonal direction; (**e**) stretching in a horizontal direction; (**f**) stretching in a lateral direction.

**Figure A3:**
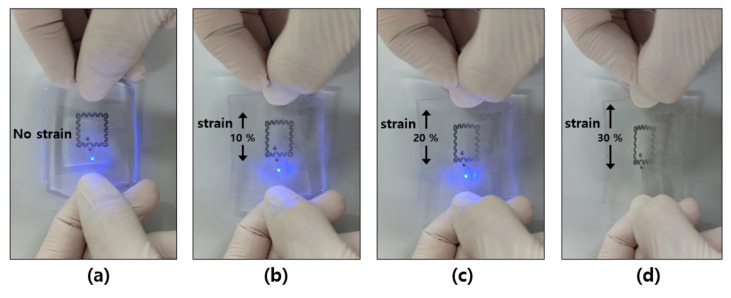
Wireless operation of stretchable optoelectronic devices with different strains: (**a**) no strain; (**b**) 10% strain; (**c**) 20% strain; (**d**) 30% strain (failure).

Unlike silicon-based electronic devices, the LED has the flexibility to be bent or stretched because it is made of PDMS, an elastomeric material, and has an antenna made of a thin aluminum film. Figure 5 shows the flexibility as it was tested in various shapes, from wrapping on a beaker with a radius of 12 mm (Figure 5a) to bending in the lateral (Figure 5b) and horizontal (Figure 5c) directions of the rectangular antenna. Wireless operation was also confirmed under the condition of twisting in a diagonal direction (Figure 5d) or stretching in the horizontal (Figure 5e) and lateral directions (Figure 5f). In the pulling test, it is evident that even with a strain of 20% each, the LED operated well without a significant decrease in its brightness, which was because the aluminum material of the serpentine-type antenna stretched as the PDMS was stretched. In its current stretchable structure, it was configured with a width of 2 mm (the difference between the antenna’s inner and outer diameters) to maintain an antenna coil of five turns, and we confirmed that it had a maximum allowable strain of about 25 to 30% based on the corresponding device structure (Figure A3).

### 3.5. Stretchable Properties of Receiving Antenna

**Figure 6:**
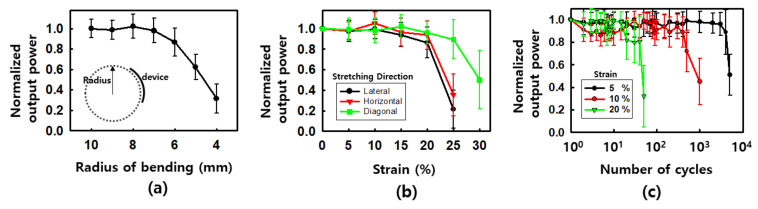
Stretchable properties of receiving antenna: (**a**) normalized output power as a function of radius of bending; (**b**) normalized output power as a function of strain; (**c**) normalized output power as a function of number of cycles.

Figure 6 shows the test results of the quantitative evaluation of bending and stretching the antenna. When bending, it was confirmed that the antenna receiving performance was noticeably weakened from about 6 mm in the radius of bending. This was not because the antenna was damaged as it stretched but because the bending radius was small compared to the size of the element, so the position of the antenna was arranged in a vertical direction rather than parallel to the transmitting antenna. Thus, the strength of the received signal was reduced. In the case of stretching, tests were conducted in the lateral, horizontal, and diagonal directions. In the horizontal and vertical directions, failure occurred in the Al pad of the antenna or LED connection part at a strain of up to 25% and in the diagonal direction up to a maximum of 30%. After 30% stretching, a dysfunctional device was placed on the transmission antenna, and it was confirmed that the LED device was turned on again by pressing the Al pad, which was confirmed in Video S1. Since the geometry of the antenna may affect the performance of the antenna, changes in the diameter or magnetic field direction of the receiving antenna due to stretching or wrapping may affect the receiving performance. However, compared to the previously reported ultra-high frequency (UHF) type, since the antenna in this study is most affected by the diameter and rotational speed, the antenna performance changes less according to various stretching situations except when wrapping and bending, in which case the direction of the antenna changes [7,21]. A fatigue test was conducted to confirm its stability against repetitive stretching, and according to the strain, it was found to maintain stable characteristics when stretched more than 3000 times in 5%, about 1000 times in 10%, and several dozen times in 20%. Although the maximum strain or stability results were slightly lower than the previously reported stretchable device, this was believed to be due to complex reasons such as a limitation of the outer and inner diameters of the antenna, an increase in overall thickness due to the multilayer antenna structure, and a low adhesion [7].

### 3.6. Applications to Optogenetics, Smartphones and Micro-Heaters

**Figure 7:**
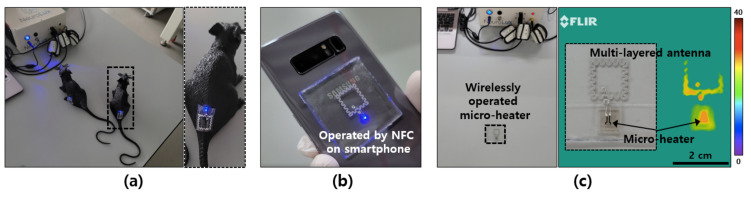
Applications on wearable and smart electronics: (**a**) attached on mouse model to show optogenetic approaches; (**b**) operated with NFC function of smartphone; (**c**) integrated with micro-heater for wireless operation of wearable heater device.

It is expected that the antenna, which can be stretched and exhibits a high wireless receiving performance at a small size, can be applied to various wearable devices. In this study, a wireless optoelectronics device for use in optogenetics, one of the latest neuroscience and neuroengineering technologies, was fabricated based on the antenna [6,7,8,21]. The device can be used to stimulate genetically modified nerves such that the LEDs are stimulated by light after implantation in the animal’s brain, spinal cord, or peripheral nerves. In Figure 7a, a mouse model placed in a large and open space was used in a real-time wireless photo-stimulation while freely moving around with an implanted optogenetic device. This showed that the optogenetic device can be operated wirelessly after attaching it to the spinal cord and near the thigh muscles of two mouse models. It is expected that in actual animal experiments, optogenetic devices inserted into moving animals in the laboratory space will stimulate nerves in real time, as reported previously [6,7,8,21]. In addition to the existing wireless power transmission system, it is also possible to drive this optoelectronic device wirelessly using the NFC function of the smartphone. This is because the rf of the smartphone NFC is 13.56 MHz, the same frequency as the device. Because of that matching frequency, it is also expected that complex control will be possible with the addition of NFC chips in the future. In addition, as shown in Figure 7c, a microheater can be connected to a wireless device instead of an LED, and an IR camera measurement can successfully verify the wireless driving of the microheater using this small receiving antenna. This stretchable, high-performance, multilayer wireless antenna can be connected to various functional elements and is expected to be used in various ways, such as in biomedical devices, wearable sensors, and smart electronics for health care or treatment.

## 4. Conclusions

Flexible and stretchable functional electronic devices, which cannot fully utilize existing silicon-based semiconductors and electronic devices, have been studied through low-performance organic semiconductor devices or silicon nanostructures requiring many processes. Recently, devices that can be bent or stretched while using existing hard semiconductor chips have been reported. In this study, a process of manufacturing high-performance multi-layered stack antennas that can be used for soft electronics was developed and applied to LEDs, microheater devices, etc. Up to six layers of aluminum antennas were introduced to confirm up to three times the performance improvement compared to the single antenna layer. Multi-layered antenna devices have a maximum thickness of 300 μm or less and have various flexibility characteristics, such as wrapping, bending, stretching, etc. The wireless receiving performance according to the antenna structure and layer was confirmed, and stability tests showed up to 30% strain and fatigue tests showed up to 3000 times at a 5% strain. In addition, the optogenetic applicability and related NFC function of smartphones was confirmed. Based on the flexibility and high-performance characteristics of this multilayer antenna process and device, we expect that it can be used in various ways, such as in soft biomedical devices applied to living bodies, optogenetics, or wearable electronic devices.

## Data Availability

Not applicable.

## Supplementary Materials

The following supporting information can be downloaded at: https://www.mdpi.com/article/10.3390/ma15093275/s1, Video S1: Device failure after stretching.

## Appendix A

Figure A1 shows photographs of the detailed fabrication process for a stretchable, multi-layered antenna device. Photographs of wirelessly operated optoelectronic devices as a function of the number of layers with different transmitting powers are presented in Figure A2. Figure A3 presents the wireless operation performances of stretchable optoelectronic devices with different strains.
